# Compression methods after femoral artery puncture: A protocol for systematic review and network meta-analysis

**DOI:** 10.1097/MD.0000000000024506

**Published:** 2021-01-29

**Authors:** Hong-Zhuo Chen, Wan-Sheng Liang, Wu-Feng Yao, Tian-Xi Liu

**Affiliations:** a Hemodialysis Center of the First Hospital of Lanzhou University; b School of Nursing, Lanzhou University; c Department of Anesthesiology, Gansu Provincial Hospital of Traditional Chinese Medicine; d Department of Nephrology, First Hospital of Lanzhou University, Lanzhou, Gansu Province, China.

**Keywords:** compression, femoral artery puncture, network meta-analysis

## Abstract

**Background:**

Vascular complications at the puncture site is a common complication after femoral artery puncture. It will not only affect the postoperative effect and patient comfort, but also may endanger the life of the patient. The effective compression hemostasis methods at the puncture site can improve the comfort of the patient, shorten the hospital stay, and reduce the burden on the medical staff. The purpose of this research is to evaluate the effectiveness and safety of different compression methods after femoral artery puncture.

**Methods:**

We will include all relevant randomized controlled trials by searching major Chinese and English databases and clinical trial registration platforms. Use Cochrane Collaboration's Risk of bias tool for bias risk analysis. Use the Grades of Recommendation, Assessment, Development, and Evaluation to assess the quality of evidence. Data analysis will be performed using Stata (V.15.0) and WinBUGS (V.1.4.3).

**Results:**

Five hundred ninety-seven records were obtained by searching the database but no records were obtained by other means. After removing duplicate records, 377 records remain. We excluded 103 records through abstract and title, leaving 274 full-text articles.

**Conclusion:**

This study will compare the application effects of different compression methods after femoral artery puncture. We hope that this study will help guide clinical decision-making and provide evidence for the management of patients after femoral artery puncture.

**Protocol Registration number:**

INPLASY2020120094.

## 1 Introduction

With the development of medical technology, interventional therapy has become the first choice for diagnosis and treatment of many diseases such as tumor, coronary heart disease, cerebrovascular disease, and peripheral vascular disease.^[1]^ Commonly used vascular puncture paths in interventional therapy are femoral artery and radial artery. Radial artery puncture is less traumatic, bleeding is easy to oppress, and does not restrict the patient's activities, but its blood vessel diameter is small, prone to spasm, and easy to rupture.^[2]^ The femoral artery has become the most commonly used puncture route for various interventional treatments due to its thick and straight, relatively fixed, obvious pulsation, and high puncture success rate.^[3]^ With the wide application of arterial puncture technology, vascular complications at the puncture site have also become the focus of clinical work. The occurrence of vascular complications will not only affect the postoperative effect and patient comfort, but also may endanger the life of the patient.^[4]^ Approximately 5% to 10% of patients will experience vascular complications after femoral artery puncture.^[5]^ The 1-year mortality rate of patients with vascular complications after puncture is 7.5%, while that of patients without complications is 1.1%.^[6]^ Among the major vascular complications, pseudoaneurysms, hematomas, arteriovenous fistulas and retroperiton -eal bleeding are mainly caused by technical problems and insufficient bleeding control.^[7]^ After the puncture, the patient's lower limbs are immobilized for a long time, and symptoms such as backache and dysuria are prone to occur.^[8]^

Therefore, an effective compression hemostasis method for the puncture site can not only reduce the occurrence of postoperative complications, improve patient comfort, and shorten the length of hospitalization, but also reduce the burden on medical staff and improve work efficiency.^[9,10]^ At present, the commonly used methods of compression hemostasis in clinical practice mainly include traditional compression methods and compression device. Among them, the traditional compression methods mainly include manual compression, bandages, sandbags and so on. They are widely used in clinical practice, have low economic costs, can effectively stop bleeding, but are time-consuming and labor-intensive.^[11]^ Compression devices mainly include arterial compressors and compression balloons. They mainly use mechanical pressure to compress the femoral artery puncture site from outside the body to promote hemostasis and healing of the puncture port. Compared with the traditional compression method, the compression device is easy to operate and saves time and effort, but its cost is high and it has not been fully used in clinical practice.^[12]^

At present, there are few systematic reviews of different compression hemostasis methods for femoral artery puncture, and the effectiveness and safety of different compression hemostasis methods are still unclear. In this study, we will conduct a systematic review and network meta-analysis (NMA) to evaluate the effectiveness and safety of femoral artery puncture with different compression methods.

## 2 Methods

### 2.1 Eligibility criteria

#### 2.1.1 Type of study

We will include all randomized controlled trials (RCTs) that compare different compression methods for femoral artery puncture, including crossover trials. There are no language restrictions.

#### 2.1.2 Type of patient

We will include all patients undergoing femoral artery puncture, regardless of their disease.

#### 2.1.3 Type of interventions

We will include RCTs that include different compression methods. For interventions, manual compression, bandages, sandbags, compression tourniquets, compression balloons, arterial compressors, etc are included.

#### 2.1.4 Type of outcomes

Primary outcomes

(1) Effectiveness includes time-to-hemostasis,^[13]^ limb braking time.
(2) Safety includes the incidence of various complications, mainly hematoma, vagus -nerve reflex, pseudoaneurysm, puncture site infection, and subcutaneous oozing, etc.^[2]^

Second outcomes

(1) Patient comfort is assessed through self-made comfort scale and patient interviews, including back pain, numbness, dysuria, etc.^[14]^
(2) Overall patient satisfaction is assessed using the satisfaction scale.^[15]^

### 2.2 Data source

We will systematically search PubMed, Embase, Web of Science, Cochrane Library, CNKI Database, VIP, Wanfang Database, and Chinese BioMedical Literature Database to identify relevant trials. We will also search major trials registries for unpublished data, including the WHO International Clinical Trials Registry Platform, Clinical Trials. Gov., Cochrane Central Register of Controlled Trials. In addition, we will track the references contained in the literature and search for other related studies through search engines (such as Google). The search terms will include “femoral artery”, “punctures”, “compress”. Detail of search strategy of PubMed is shown in Table 1.

**Table 1:** Searching strategy in PubMed.

#1 “Femoral Artery”[MeSH]
#2 “Femoral artery”[title/abstract] or “femoral arteries”[title/abstract]
#3 #1 or #2
#4 “Punctures”[MeSH]
#5 “Punctur^∗^”[title/abstract]
#6 #4 or #5
#7 “Compression bandages”[MeSH] or “stockings, compression”[MeSH]
#8 “Compression”[title/abstract]
#9 #7 or #8
#10 “Clinical trials, phase II as topic”[MeSH] or “clinical trials, phase III as topic”[Mesh] or “clinical trials, phase IV as topic”[MeSH] or “controlled clinical trials as topic”[MeSH] or “randomized controlled trials as topic”[MeSH] or “intention to treat analysis”[MeSH] or “pragmatic clinical trials as topic”[mesh] or “clinical trials, phase II”[publication type] or “clinical trials, phase III”[publication type] or “clinical trials, phase iv”[publication type] or “controlled clinical trials”[publication type] or “pragmatic clinical trials as topic”[publication type] or “single-blind method”[MeSH] or “double-blind method”[MeSH]
#11 Random^∗^[title/abstract] or blind^∗^[title/abstract] or singleblind^∗^[title/abstract] or doubleblind^∗^[title/abstract] or trebleblind^∗^[title/abstract] or tripleblind [title/abstract]
#12 #10 or #11
#13 #3 and #6 and #9 and #12

### 2.3 Selection of trials

We will use Endnote X8 (Clarivate Analytics) to manage all citations from the database, and 2 independent reviewers (HZC and WSL) will screen the citations by title and abstract. Then, we will obtain the full texts for further evaluation. Two independent reviewers (WSL and WFY) will extract data from the included studies, including research characteristics (first author name, publication year, journal), patient characteristics (age, sample size), intervention and outcome, etc. Any disagreements will be resolved by a third review author.

### 2.4 Risk of bias analysis

Evaluate the methodological quality of RCTs according to the Cochrane Bias Risk Assessment Tool (Cochrane Intervention Manual for Systematic Review). The tool consists of 6 domains^[16]^:

1. sequence generation,
2. allocation concealment,
3. blinding of participants and personnel,
4. blinding of outcome assessors,
5. incomplete outcome data and selective outcome reporting,
6. and other sources of bias.

We will evaluate methodological quality as “low risk”, “unclear risk”, or “high risk”.^[17]^ The assessment process is carried out by 2 reviewers independently (TXL and WFY), and a third investigator will resolve any differences.

### 2.5 Statistical analysis

#### 2.5.1 NMA

We will use the random effect model of Stata V.15.0 (Stata Corporation, College Station, TX)^[18]^ to conduct a paired meta-analysis of direct evidence. Dichotomous data will be expressed as relative risk with 95% confidence interval, and continuous results will be expressed as standard mean difference with 95% confidence interval. Statistical heterogeneity will be checked using I^2^ statistics and *P* value. If *P* value < .1 and I^2^ > 50%, the study is considered to be heterogeneous. We will explore the source of heterogeneity through sensitivity analysis and subgroup analysis.^[19]^ In order to compare the effectiveness of different compression methods for patients after femoral artery puncture, we will perform NMA. NMA combines direct and indirect evidence for all relative treatment effects and provides estimates with maximum power. We will use the Markov Chain Monte Carlo in WinBUGS V.1.4.3 (MRC Biostatistics Unit, Cambridge, UK)^[20]^ to perform random effects NMA within the Bayesian framework. Three Markov chains will be used for simulation, and the number of iterations will be set to 50,000. We will also rank the effects of different interventions and record the area under the curve, the area under the curve is expressed as a percentage, the larger the value, the better the effect.^[21]^

#### 2.5.2 Subgroup analysis and sensitivity analysis

Subgroup analysis will be considered if sufficient data is available, such as differences between sexes, age of participants, and comparison between different countries. In addition, we will conduct sensitivity analysis by excluding low-quality studies and trials with imputed missing data.

### 2.6 Quality of evidence

Grading of Recommendations Assessment, Development, and Evaluation framework^[22]^ will be used to assess the quality of evidence in NMA, which characterises the quality of a body of evidence on the basis of the study limitations, imprecision, heterogeneity or inconsistency, indirectness and publication bias. The quality of the evidence will be assessed as “high”, “moderate”, “low”, or “very low”.^[23]^

## 3 Result

### 3.1 Results of the search

**Figure 1:**
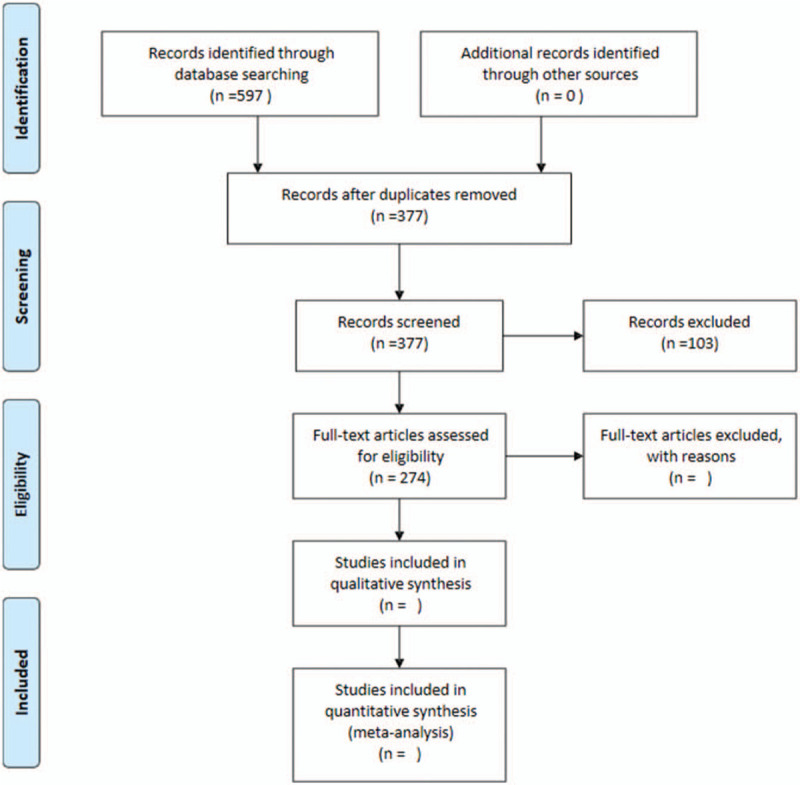
The flowchart of the screening process.

Five hundred ninety-seven records were obtained by searching the database but no records were obtained by other means. After removing duplicate records, 377 records remain. We excluded 103 records through abstract and title, leaving 274 full-text articles. The document screening flowchart is shown in Figure 1.

### 3.2 Characteristic of included studies

In a preliminary trial, we included 8 studies. The average age of patients was 51 to 66, with a maximum sample size of 964 and a minimum sample size of 128. For more detailed information, see Table 2.

**Table 2:** Basic characteristics of some of the included studies.

				Interventions		
Author Yr	Age	Gender (male /female)	Sample (Experimental /Control)	Experimental group	Controlled group	Outcomes
Hermanides, R. S2010^[24]^	64.5 ± 11.3	476/151	313/314	Vascular closure devices	MC	Complications (bleeding; hematoma; arteriovenous fistula)
Hallak, O.K. 2007^[15]^	No report	218/158	187/189	The D-STAT Dry hemostatic bandage + MC	MC	TTH; Complications (pseudo-aneurysm; rebleed; hematoma); Overall patient satisfaction
Holm, N.R. 2014^[13]^	64.7 ± 11	621/343	483/481	FemoSeal vascular closure devices	MC	TTH; Complications (major bleeding; pseudoaneurysm; infection; retroperitoneal bleeding; arteriovenous fistula)
Trabattoni, D. 2011^[7]^	66 ± 11	130/70	100/100	Haemostatic bandage	MC	TTH; Complications (pseudoaneurysm; haematoma; retroperitoneal bleeding; major bleeding)
Botti, M. 1998^[14]^	61.4 ± 11.2	737/313	556/519	MC+Pressure bandage	MC	TTH; Complications (bruising); Patient comfort
ZhouJ 2012^[25]^	51 ± 5	198/42	120/120	Arterial compressor	MC+bandage+sandbag	TTH; Limb braking time; Complications (skin blisters and damage; petechiae; hematoma); Patient comfort
Zuo YK 2011^[2]^	53.2 ± 3.8	90/84	81/93	Airbag compression	MC+sandbag	Limb braking time; Complications (venous thrombosis of lower limbs; pseudoaneurysm; infection; hematoma; vagal reflex; subcuta -neous hemorrhage); Patient comfort
Ma Q 2011^[26]^	No report	93/35	64/64	Arterial compression tourniquet	MC+sandbag	TTH; Complications (pseudoaneurysm; hematoma; skin ecchymosis; hemorrhage) Limb braking time

MC = manual compression, TTH = time-to-hemostasis.

## 4 Discussion

At present, there is no relevant NMA to compare the application effects of different compression hemostasis methods after femoral artery puncture. Therefore, this systematic review and NMA will summarize the direct and indirect comparative evidence to evaluate different methods of compression hemostasis. We hope that this study will help guide clinical decision-making and provide evidence for the management of patients after femoral artery puncture.

## Author contributions

Hong-Zhuo Chen drafted this protocol and developed the search strategies. Wan-Sheng Liang and Wu-Feng Yao contributed to the extraction of research data. Tian-Xi Liu and Wu-Feng Yao contributed to evaluation of bias. Wan-Sheng Liang contributed analysis of results. Tian-Xi Liu evaluated of bias and approved the final manuscript. All authors approved the final version of the manuscript.

**Conceptualization:** Hong Zhuo Chen.

**Data curation:** Wan Sheng Liang, Wu Feng Yao.

**Formal analysis:** Wan Sheng Liang, Wu Feng Yao.

**Methodology:** Hong Zhuo Chen.

**Project administration:** Tian xi Liu.

**Software:** Wu Feng Yao.

**Writing – original draft:** Hong Zhuo Chen, Tian xi Liu.

**Writing – review & editing:** Tian xi Liu.

## Abbreviations

Abbreviations: NMA = network meta-analysis, RCTs = randomized controlled trials.
